# Impact of chronic kidney disease on the incidence of visual impairment and age-related eye diseases in a multi-ethnic Asian population

**DOI:** 10.7189/jogh.15.04316

**Published:** 2025-11-28

**Authors:** Yulia Liem, Vishnu Vemula, Cynthia Ciwei Lim, Crystal Chun Yuen Chong, Jason Chon Jun Choo, Ching-Yu Cheng, Charumathi Sabanayagam

**Affiliations:** 1 Singapore Eye Research Institute, Singapore National Eye Centre, Singapore; 2 Lee Kong Chian, School of Medicine, Nanyang Technological University, Singapore; 3 Department of Renal Medicine, Singapore General Hospital, Singapore; 4 Department of Ophthalmology, Yong Loo Lin School of Medicine, National University of Singapore, Singapore; 5 Centre for Innovation and Precision Eye Health, Yong Loo Lin School of Medicine, National University of Singapore, Singapore; 6 Ophthalmology and Visual Science Academic Clinical Programme, Duke-NUS Medical School, Singapore

## Abstract

**Background:**

The kidney and eye share common metabolic and vascular risk factors, and chronic kidney disease (CKD) has been associated with the prevalence of visual impairment (VI). In this study, we examined the association of CKD with incident VI and major age-related eye diseases, including cataract, age-related macular degeneration (AMD), diabetic retinopathy (DR), and glaucoma, in a multi-ethnic Asian population.

**Methods:**

We analysed data from 6486 Chinese, Malay, and Indian adults aged 40–80 years who participated in the Singapore Epidemiology of Eye Diseases study at baseline (2004–11) and six-year follow-up visit (2011–17) and were free of VI and the respective eye diseases at baseline. We defined CKD (n = 564; 8.7%) as an estimated glomerular filtration rate (eGFR)<60 ml/min/1.73 m^2^, and categorised the severity of CKD into stages G1–G5. Eye examinations included refraction, slit-lamp examinations, and retinal imaging. We defined incident VI as best-corrected visual acuity <20/40 in the better eye. Eye diseases examined included cataract, AMD, retinopathy, including DR in those with diabetes and glaucoma. We examined associations between CKD, VI, and eye diseases using multivariable logistic regression models adjusted for age, gender, ethnicity, diabetes, and hypertension status, presenting the results as odds ratios (ORs) and 95% confidence intervals (CIs).

**Results:**

CKD participants had a higher incidence of any VI (14.3% *vs.* 3.3%; *P* < 0.001), any AMD (8.0% *vs.* 5.4%; *P* < 0.001), and cataracts (65.1% *vs.* 40.8%; *P* < 0.001) than non-CKD participants. VI incidence increased with CKD severity in G1–G2 (3.3%), G3a (13.5%), and G3b–G5 (16.3%) (*P* < 0.001). In multivariable models, CKD was associated with incident VI (OR = 1.47; 95% CI = 1.03–2.10) and moderate/worse DR (OR = 2.62; 95% CI = 1.35–5.10).

**Conclusions:**

Our results suggest that the presence of CKD increases the risk and severity of VI and eye diseases in Asian adults. Our findings highlight the importance of regular eye exams for CKD patients to reduce the risk of VI.

Chronic kidney disease (CKD) remains one of the major healthcare challenges worldwide associated with an increased risk of cardiovascular disease, renal failure, and poor quality of life. In the USA alone, it is estimated that in 2023, one in seven people have CKD, with 90% of them being unaware of their condition [1]. In Singapore, based on the national population health survey, CKD increased from 8.7% in 2019–20 to 13.8% in 2021–22 [2,3].

Age-related eye diseases, including cataract, diabetic retinopathy (DR), age-related macular degeneration (AMD), and glaucoma, are major causes of blindness and vision impairment (VI) in middle-aged and older adults, leading to reduced quality of life and strain on healthcare systems globally [4]. Eye and kidney diseases share common risk factors, including diabetes, hypertension, and smoking, as well as mechanisms like oxidative stress and inflammation [5]. Several cross-sectional studies have shown that the burden of VI and age-related eye diseases is significantly higher in those with CKD compared to the general population [6–8]. The prevalence of CKD is expected to continue increasing, driven primarily by population growth, ageing, and the rising prevalence of diabetes, heart disease, and hypertension. With the increasing prevalence of CKD, the prevalence of age-related eye diseases is also expected to rise.

While several cross-sectional studies have identified associations between CKD and VI, cataracts, DR, and glaucoma [7–9], there is a lack of comprehensive prospective research exploring the incidence of these eye diseases in CKD patients. In our previous study, we found that the presence of CKD was significantly associated with VI, cataract, retinopathy, and DR [6]. Few prospective studies have assessed the association between CKD and the incidence of specific eye diseases. For instance, CKD has been shown to be associated with increased risk of cataract in a Chinese population in Taiwan [10] and with the increased risk of AMD in studies conducted in the USA [10-12]. However, no study has assessed the impact of CKD on the incidence of VI and other age-related blinding eye diseases.

We aim to fill this gap by examining the association of CKD with the incidence of VI and major age-related eye diseases such as DR, AMD, glaucoma, and cataracts in a multi-ethnic Asian population.

## METHODS

### Data set

We derived the data from the Singapore Epidemiology of Eye Diseases study, a population-based cohort study of 10 033 Chinese, Malay, and Indian adults aged 40–80 years at baseline [13]. The study evaluated the prevalence, incidence, progression, and risk factors of age-related eye diseases and VI and was carried out sequentially and independently among Singapore's three major ethnic groups: Malays (Singapore Malay Eye Study, 2004–06 and 2011–13), Indians (Singapore Indian Eye Study, 2007–09 and 2013–15), and Chinese (Singapore Chinese Eye Study, 2009–11 and 2015–17). All these studies were conducted at the same research clinic, the Singapore Eye Research Institute, and followed similar protocols. Participants were recruited following an age-stratified random sampling at baseline. All recruited participants provided written informed consent before completing standard interviewer-administered questionnaires (including demographic information and medical history), ocular examinations (retinal photography and visual field assessments), and venous blood collection for laboratory testing. We followed the STROBE reporting guidelines for cohort studies (Table S1 in the **Online Supplementary Document**) [14].

### Inclusion and exclusion criteria

**Figure 1:**
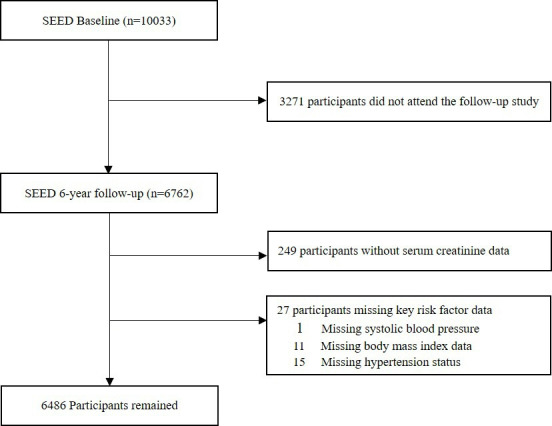
Flowchart of participant inclusion and exclusion criteria.

During the six-year follow-up, 3271 individuals did not attend. For the current analysis, we included 6762 participants who attended both baseline (2004–11) and six-year follow-up visits (2011–17) (**Figure 1**). Of the 6762 participants, we included 6486 participants for the final analysis after excluding participants who had missing information on serum creatinine at baseline (n = 249) and other key variables, including systolic blood pressure (BP), body mass index (BMI), and hypertension (n = 27).

For each outcome, we excluded participants with the condition at baseline, and included the remaining in the respective incident analyses. For example, for outcome ‘Any VI’, after excluding those with prevalent VI, we included 4732 participants; final sample size after excluding respective prevalent conditions for individual eye diseases were 3379 for any eye disease, 5753 for any retinopathy, 5893 for AMD, 6299 for glaucoma, 3799 for cataract, and 3918 for under-corrected refractive errors (URE). There were 3426 participants with diabetes at baseline. After excluding those with prevalent DR, we included 1262 participants for incident DR and 1507 for incident moderate/worse DR analysis.

### Assessment and definition of VI and eye diseases

We defined VI as best-corrected visual acuity (BCVA)<20/40 in the better eye. Based on BCVA in the better eye, we categorised the severity of VI into normal vision (BCVA≥20/40, logMAR≤0.30), low vision (20/40<BCVA<20/200, logMAR = 0.30–1.00), and blindness (BCVA≤20/200, logMAR≥1.00). We assessed retinopathy, DR (in those with diabetes), AMD, glaucoma, cataract, and URE as eye diseases. We considered any retinopathy to be present if characteristic lesions, as defined by the Early Treatment DR study, were noted on retinal photographs (Table S2 in the **Online Supplementary Document**). We defined DR as the presence of retinopathy in those with diabetes, defining levels as no DR (DR<20), minimal DR (DR = 20), mild DR (DR = 35), moderate DR (DR = 43–47), severe DR (DR = 53), and proliferative DR (DR = 61–90). We defined AMD as the presence of either early or late AMD based on fundus photographs graded according to the Wisconsin Age-Related Maculopathy Grading System. We defined glaucoma as the presence of both glaucomatous visual field loss and optic disk changes in one or both eyes, defining glaucomatous visual field loss as a glaucoma hemifield test graded ‘outside normal limits’ and a cluster of three contiguous points at the 5% level on the pattern deviation plot. We defined cataract as the presence of nuclear, cortical, or posterior subscapular cataract using the Lens Opacities Classification System III cataract grading system or history of cataract surgery. We defined URE as an improvement of at least 0.2 logMAR (equivalent to two lines) in BCVA relative to the presenting visual acuity in the better eye. We defined the incidence of each outcome (VI, any retinopathy, AMD, glaucoma, cataract, and URE) as the proportion of participants who were free of that outcome at baseline and developed it at follow-up. For example, we defined incident VI as the occurrence of VI at follow-up, among those who were free of VI at baseline. Moreover, we defined incident moderate/worse DR as the presence of moderate, severe, or proliferative DR at follow-up in participants with diabetes who were free of moderate/ worse DR at baseline. We also included a composite outcome, ‘any eye disease’, defined as the occurrence of any of the above eye diseases (any retinopathy, AMD, glaucoma, and cataract) at follow-up in those free of these diseases at baseline.

### Assessment of CKD

We defined CKD as an estimated glomerular filtration rate (eGFR)<60 ml/min/1.73 m^2^, calculated from serum creatinine using the CKD Epidemiology Collaboration equation [15]. We defined CKD severity as stage G1, G2 (eGFR ≥ 60 mL/min/1.73 m^2^), stage G3a (eGFR = 45–60 mL/min/1.73 m^2^), stage G3b (eGFR = 30–45 mL/min/1.73 m^2^), stage G4 (eGFR = 15–30 mL/min/1.73 m^2^), and stage G5 (eGFR<15 mL/min/1.73 m^2^). For analysis, we collapsed stages G3b, G4, and G5 into a single category due to the small number of cases (n = 160) in G4 and G5.

### Assessment of covariates

We used a detailed interviewer-administered questionnaire to collect demographic and medical history data from all participants. We obtained information on diabetes and hypertension from self-report, physical examination, and laboratory examination [6]. We obtained the blood samples and analysed them for total cholesterol, high-density lipoprotein cholesterol, and low-density lipoprotein cholesterol, glycosylated haemoglobin A1c (HbA1c), creatinine, and random glucose. We defined diabetes as random glucose ≥11.1 mmol/L, HbA1c% ≥ 6.5%, use of diabetic medication, or self-reported history of physician-diagnosed diabetes mellitus. We measured BP for all participants with an automatic BP monitor (Dinamap model Pro Series DP110X-RW, 100V2; GE Medical Systems Information Technologies Inc., Milwaukee, USA) twice, five minutes apart. If systolic readings differed by >10 mm Hg or diastolic readings by >5 mm Hg, we took a third measurement. We recorded the average of the two closest readings as the participant’s BP value [16]. Lastly, we defined hypertension as systolic BP≥140 mm Hg, diastolic BP≥90 mm Hg, use of antihypertensive medication, or self-reported history of physician-diagnosed hypertension.

### Statistical analysis

We assessed the normality of continuous variables using the Shapiro-Wilk test and histograms. We summarised the population characteristics for those with and without baseline CKD as mean (standard deviation (SD)) for normally distributed continuous variables and number (%) for categorical variables. We determined the corresponding *P*-values using Student’s *t* test, Pearson χ^2^ test, or Fisher's exact test as appropriate for each variable. We calculated the incidence as the number of new cases during follow-up divided by the number of participants at risk at baseline and presented it as bar charts. We assessed the incidence of VI across CKD severity categories using the Cochran-Armitage test for trend. We evaluated the association of baseline CKD with incident eye diseases using two logistic regression models: a simple model adjusted for age and sex, and a multivariable model additionally adjusted for ethnicity, diabetes, and hypertension status. We did not adjust for other covariates (*e.g.* smoking and BMI) because the number of events for some outcomes was insufficient to allow adjustment for multiple factors. For incident DR within the diabetic subgroup, we also included diabetes duration and HbA1c in the multivariable model. We also evaluated the associations of VI and eye diseases within each ethnic group. In a supplementary analysis, we assessed the association between baseline eGFR (per 5 mL/min/1.73 m^2^ decrease) and incident VI and ocular diseases using the same logistic regression models. We defined statistical significance as *P* < 0.05. We used *R*, version 4.0.2 (R Core Team, Vienna, Austria) for all analyses.

## RESULTS

Of the 6486 participants, 564 (8.7%) had CKD at baseline, with 23.4% Chinese, 54.8% Malays, and 21.8% Indians (**Table 1**).

**Table 1:** Characteristics of participants by baseline CKD status

	Overall (n = 6486)	CKD absent (n = 5922)	CKD present (n = 564)	*P*-value
Age, in years*	57.29 (9.42)	56.40 (8.99)	66.61 (8.82)	<0.001
**Female†**	3343 (51.5)	3082 (52.0)	261 (46.3)	0.01
**Ethnicity†**				<0.001
Chinese	2553 (39.4)	2421 (40.9)	132 (23.4)	
Indian	2119 (32.7)	1996 (33.7)	123 (21.8)	
Malay	1814 (28.0)	1505 (25.4)	309 (54.8)	
**Current smokers†**	943 (14.5)	877 (14.8)	66 (11.7)	0.05
**Alcohol drinkers†**	584 (9.0)	561 (9.5)	23 (4.1)	<0.001
**Primary/below educated†**	3571 (55.1)	3139 (53.0)	432 (76.6)	<0.001
**CVD†**	552 (8.5)	442 (7.5)	110 (19.5)	<0.001
**Hypertension†**	3849 (59.3)	3337 (56.3)	512 (90.8)	<0.001
BP, in mm Hg*				
Systolic	137.34 (20.56)	136.24 (19.95)	148.85 (23.16)	<0.001
Diastolic	78.18 (10.26)	78.14 (10.16)	78.53 (11.24)	0.4
BMI, in kg/m^2^*	25.36 (4.44)	25.26 (4.44)	26.42 (4.32)	<0.001
**Hyperlipidaemia†**	2877 (44.6)	2523 (42.8)	354 (63.2)	<0.001
Blood cholesterol, in mmol/l*				
Total	5.42 (1.08)	5.43 (1.07)	5.32 (1.24)	0.02
LDL	3.38 (0.93)	3.40 (0.92)	3.20 (1.01)	<0.001
HDL	1.24 (0.37)	1.25 (0.37)	1.22 (0.33)	0.08
**Diabetes mellitus†**	1713 (26.4)	1450 (24.5)	263 (46.6)	<0.001
Blood glucose, in mmol/l*	6.62 (3.07)	6.53 (2.98)	7.51 (3.83)	<0.001
**Medication use†**				
Anti-hypertensive	2110 (54.8)	1748 (52.4)	362 (70.7)	<0.001
Anti-diabetes	1041 (60.8)	864 (59.6)	177 (67.3)	0.02
Anti-cholesterol	1569 (24.3)	1313 (22.3)	256 (45.7)	<0.001
eGFR, in ml/min/1.73m^2^*	86.13 (18.16)	89.70 (14.29)	48.62 (10.25)	<0.001

BMI – body mass index, BP – blood pressure, CVD – cardiovascular disease, eGFR – estimated glomerular filtration rate, HDL – high density lipoprotein, LDL – low density lipoprotein

*Values presented as mean (standard deviation).

†Values presented as number (percentage).

Compared to those without CKD, participants with CKD were older, more likely to be males, of Malay ethnicity, alcohol drinkers and had lower educational attainment (primary school or below). They also had a higher prevalence of cardiovascular disease, hypertension, hyperlipidaemia, diabetes and were more likely to be on anti-hypertensive, anti-diabetic and anti-cholesterol medications. In addition, participants with CKD had higher systolic BP, BMI, total and low-density lipoprotein cholesterol, HbA1c, and blood glucose levels (all *P* < 0.05), and lower eGFR than those without CKD.

### Incidence of VI and major age-related eye diseases in those with CKD

**Figure 2:**
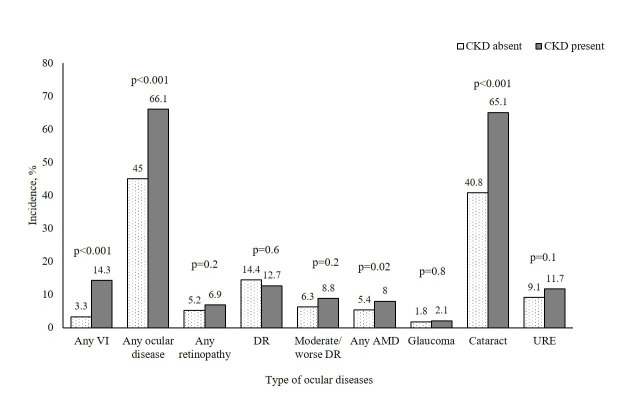
Incidence of eye diseases stratified by baseline CKD status. *P*-values were calculated using Pearson χ^2^ test and Fisher exact test for those with subgroup numbers <5. AMD – age-related macular degeneration, CKD – chronic kidney disease, DR – diabetic retinopathy, URE – under-corrected refractive error, VI – vision impairment.

The incidence of VI was more than four times higher in those with CKD compared to those without (14.3% *vs.* 3.3%; *P* < 0.001) (**Figure 2**).

Among individual eye diseases, the incidence of any eye disease (66.1% *vs.* 45%), AMD (8.0% *vs.* 5.4%), and cataract (65.1% *vs.* 40.8%) was significantly higher in those with CKD than in those without CKD. There was a clear trend of increasing incidence of VI with CKD severity, with rates of 3.3% in stage G1–G2, 13.5% in G3a, and 16.3% G3b–G5 (*P* < 0.001) (Figure S1 in **Online Supplementary Document**). Among the eye diseases, the incidence of any eye disease increased from 45.0% in G1–G2 to 63.6% in G3a and 72.7% in G3b–G5, while cataract incidence rose from 40.8% in G1–G2 to 64.0% in G3a and 68.3% in G3b–G5 (*P* < 0.001). The incidence of AMD increased from 5.4% in G1–G2 to 8.0% in G3a and 8.1% in G3b–G5 (*P* = 0.06), while URE rose from 9.1% in G1–G2 to 10.2% in G3a and 15.3% in G3b–G5 (*P* = 0.07) (Table S3 in **Online Supplementary Document**). While differences between CKD stages were not statistically significant for AMD and URE, the overall trends for AMD (*P* = 0.02) and URE (*P* = 0.03) were significant.

In the ethnicity-specified analysis, the incidence of VI was significantly higher in individuals with CKD compared to those without CKD among Chinese (16.0% *vs.* 7.2%) and Malay (15.9% *vs.* 4.2%) participants. Although a 10.1% difference was observed in Indians (12.0% *vs.* 1.9%), this difference was not statistically significant (Figure S2 in the **Online Supplementary Document**).

### Association of CKD with VI and major age-related eye diseases

CKD was independently associated with an increased risk of any VI in both age- and sex-adjusted and multivariable models (odds ratio (OR) = 1.47; 95% confidence interval (CI) = 1.03–2.10) (**Table 2**).

**Table 2:** Association of baseline CKD status with incidence of eye diseases

	At-risk individuals	CKD participants at risk	non-CKD participants at risk	Age, sex adjusted		Multivariable	
	**n (%)**	**n (%)**	**n (%)**	**OR (95% CI)**	***P*-value**	OR (95% CI)*	***P*-value**
**Any VI**	4732 (4.4)	447 (14.3)	4285 (3.3)	2.00 (1.41–2.82)	<0.001	1.47 (1.03–2.10)	<0.001
**Any eye disease**	3379 (45.8)	121 (66.1)	3258 (45.0)	1.63 (1.10–2.42)	<0.001	1.05 (0.68–1.62)	0.8
**Any retinopathy**	5753 (5.3)	451 (6.9)	5302 (5.2)	1.37 (0.91–2.06)	0.1	1.12 (0.73–1.72)	0.6
**DR†**	1262 (14.2)	173 (12.7)	1089 (14.4)	1.10 (0.66–1.82)	0.7	1.24 (0.71–2.17)	0.5
**Moderate/worse DR†**	1507 (6.6)	204 (8.8)	1303 (6.3)	1.91 (1.07–3.39)	<0.001	2.62 (1.35–5.10)	<0.001
**Any AMD**	5893 (5.6)	473 (8.0)	5420 (5.4)	0.84 (0.58–1.22)	0.4	0.74 (0.51–1.09)	0.1
**Glaucoma**	6299 (1.8)	535 (2.1)	5764 (1.8)	0.62 (0.32–1.20)	0.2	0.66 (0.34–1.29)	0.2
**Cataract**	3799 (41.7)	152 (65.1)	3647 (40.8)	1.88 (1.32–2.67)	<0.001	1.14 (0.78–1.68)	0.5
**Under-corrected refractive error**	3918 (9.3)	385 (11.7)	3533 (9.1)	1.17 (0.82–1.66)	0.4	0.96 (0.66–1.39)	0.8

AMD – age-related macular degeneration, CI – confidence interval, DR – diabetic retinopathy, CKD – chronic kidney disease, OR – odd ratio, URE – under-corrected refractive error, VI – vision impairment

*Adjusted for age, gender, ethnicity, diabetes status, and hypertension.

†Adjusted for age, gender, ethnicity, diabetes duration, and HbA1c.

Among the eye diseases, a significant association was observed only with moderate/worse DR (OR = 2.62; 95% CI = 1.35–5.10). While CKD was associated with any eye disease and cataract in age- and sex-adjusted models, these associations lost significance in multivariable models. No significant associations were observed between CKD and other outcomes, including any retinopathy, any AMD, or glaucoma, in either age- and sex-adjusted or multivariate models.

In supplementary analyses using eGFR modelled as a continuous variable, lower baseline eGFR (per 5 ml/min/1.73 m^2^ decrease) was associated only with incident VI in multivariable models (OR = 1.06; 95% CI = 1.01–1.11) (Table S4 in the **Online Supplementary Document**).

In the analysis stratified by ethnicity (**Table 3**), the association between CKD and VI was positive but not statistically significant across the three ethnic groups.

**Table 3:** Association of baseline CKD status with incidence of eye diseases stratified by ethnicity

	Indian				Chinese				Malay			
	**CKD participants at risk**	**Non-CKD participants at risk**	**Multivariable**		**CKD participants at risk**	**Non-CKD participants at risk**	**Multivariable**		**CKD participants at risk**	**Non-CKD participants at risk**	**Multivariable**	
	**n (%)**	**n (%)**	OR (95% CI)*	***P*-value**	**n (%)**	**n (%)**	OR (95% CI)*	***P*-value**	**n (%)**	**n (%)**	OR (95% CI)*	***P*-value**
**Any VI**	50 (16.0)	483 (7.2)	1.17 (0.47–2.89)	0.7	120 (10.0)	2361 (1.9)	1.35 (0.64–2.84)	0.4	277 (15.9)	1441 (4.2)	1.52 (0.96–2.41)	0.07
**Any eye disease**	16 (75.0)	1052 (63.5)	0.92 (0.28–3.01)	0.9	29 (72.4)	1353 (24.8)	3.69 (1.49–9.14)	<0.001	76 (61.8)	853 (54.2)	0.61 (0.35–1.04)	0.08
**Any retinopathy**	75 (12.0)	1687 (8.2)	1.22 (0.57–2.64)	0.6	123 (6.5)	2240 (2.8)	1.85 (0.80–4.28)	0.2	253 (5.5)	1375 (5.4)	0.86 (0.45–1.65)	0.6
**DR†**	39 (17.9)	480 (18.1)	1.69 (0.66–4.31)	0.3	31 (12.9)	300 (8.0)	2.46 (0.68–8.87)	0.2	103 (10.7)	309 (14.9)	0.81 (0.36–1.85)	0.6
**Moderate/worse DR†**	58 (15.5)	626 (6.9)	7.19 (2.66–19.43)	<0.001	33 (12.1)	337 (4.5)	7.74 (1.68–35.69)	<0.001	113 (4.4)	340 (7.1)	0.53 (0.16–1.73)	0.3
**Any AMD**	101 (5.9)	1824 (5.5)	0.49 (0.20–1.19)	0.1	108 (13.0)	2208 (4.7)	1.37 (0.72–2.61)	0.3	264 (6.8)	1388 (6.5)	0.66 (0.37–1.16)	0.2
**Glaucoma**	120 (4.2)	1961 (2.7)	0.86 (0.32–2.28)	0.8	123 (4.1)	2349 (1.3)	1.56 (0.54–4.50)	0.4	292 (0.3)	1454 (1.5)	0.09 (0.01–0.71)	<0.001
**Cataract**	27 (81.5)	1225 (59.8)	1.51 (0.55–4.16)	0.4	34 (64.7)	1497 (19.6)	3.04 (1.35–6.84)	<0.001	91 (60.4)	925 (49.9)	0.67 (0.41–1.09)	0.1
**URE**	44 (20.5)	338 (29.3)	0.82 (0.35–1.86)	0.6	104 (3.8)	1975 (5.4)	0.49 (0.17–1.41)	0.2	237 (13.5)	1220 (9.4)	1.08 (0.68–1.71)	0.7

AMD – age-related macular degeneration, CI – confidence interval, DR – diabetic retinopathy, CKD – chronic kidney disease, OR – odd ratio, URE – under-corrected refractive error, VI – vision impairment

*Multivariable logistic regression models adjusted for age, gender, diabetes status, and hypertension.

†Multivariable model additionally adjusted for diabetes duration and HbA1c.

Among eye diseases, CKD was significantly associated with the incidence of any eye disease, moderate/worse DR and cataract in Chinese participants. In Indians, a significant association was observed only with moderate/worse DR. In Malays, CKD was not positively associated with any eye disease but was inversely associated with glaucoma.

## DISCUSSION

In a population-based sample of multi-ethnic Asian adults, we found that the incidence of VI and major eye diseases, including any eye disease, AMD and cataract, was significantly higher in persons with CKD compared to those without. The incidence of VI increased with the severity of CKD. In multivariable models, CKD was independently associated with the incidence of VI and moderate/worse DR (in those with diabetes), independent of potential confounding factors. In ethnicity-specific analysis, CKD was associated with any eye disease and cataract in Chinese, while incident moderate/worse DR showed significant associations with CKD in both Indians and Chinese. In Malays, CKD was inversely associated with glaucoma. To our knowledge, we are the first to report the incidence of VI and a range of age-related eye diseases among individuals with CKD.

We found that the incidence of VI was over four times higher in those with CKD compared to those without. In multivariable models, CKD was significantly associated with incident VI. Our findings support those of our previous cross-sectional study, which showed that the prevalence of VI was three times higher in individuals with CKD compared to those without [6]. These results are also consistent with findings from other studies, including the National Health and Nutrition Examination Survey in the USA, which reported a 7-fold higher prevalence of VI in individuals with CKD [17]. In addition, we observed that the incidence of VI increased with CKD severity, suggesting that more advanced stages of CKD may accelerate VI progression.

Among individual eye diseases, we found that the incidence of any eye disease, AMD and cataract was significantly higher in those with CKD. Deva and colleagues have shown that patients with CKD stages 3–5 had significantly higher rates and severity of vision-threatening retinal abnormalities such as moderate-severe microvascular retinopathy, proliferative DR, and late-stage AMD compared to patients with CKD stages 1–2, suggesting that progressive renal dysfunction is an independent risk factor for eye disease [18]. While the Blue Mountains Eye Study did not find a significant association between renal function and the incidence of specific cataract subtypes, it reported increased odds of cataract surgery among adults aged <60 years with moderate to severe renal impairment, suggesting a potential link between reduced renal function and surgical intervention in younger patients [19]. Similarly, several studies have reported a higher incidence of cataract surgeries among CKD patients undergoing dialysis, with an increased risk up to 1.8-fold overall and a 5-fold risk in those aged <60 years, even after adjusting for diabetes and hypertension [20,21]. These studies suggest that the increased risk of cataract formation may be attributed to systemic factors commonly associated with CKD, such as hypertension, diabetes, and cardiovascular disease. A Swedish case–control study further found that individuals with CKD had a significantly higher risk of developing cataracts, retinal vascular occlusions, DR, and other retinal disorders, including AMD, with disease onset occurring earlier than in those without CKD [22].

In multivariable models, we found that CKD was significantly associated with the incidence of moderate/worse DR, confirming our previous cross-sectional study findings [6]. Although the incidence of AMD and cataract was higher in those with CKD, they were not associated with CKD after adjustment for potential confounders. Several population-based studies, such as the Beaver Dam Eye Study in the USA and the Blue Mountains Eye Study in Australia, have shown that CKD is independently associated with AMD [11,12]. DR is a complication of diabetes and 46% had diabetes in our CKD population, suggesting that coexistence of CKD and diabetes significantly increases the risk of developing DR. It was assumed that the detrimental effect of hyperglycaemia, endothelial dysfunction, chronic inflammation and poor control of blood glucose levels could have played a central role in the generation of reactive oxidative species by angiotensin II, leading to inflammation and damage to retinal blood vessels [23]. Chronic inflammation in CKD contributes to endothelial dysfunction, increasing retinal vascular permeability and promoting DR. Up-regulation of transforming growth factor β plays a key role in proliferative DR by reducing VE-Cadherin and Claudin-5 expression [24,25]. Additionally, the accumulation of advanced glycation end products in CKD exacerbates retinal damage through oxidative stress and inflammation [26].

In ethnicity-specific analysis, we found CKD to be significantly associated with DR in Chinese and Indians and any eye disease and cataract in Chinese. Although Malays had a higher prevalence of CKD, no significant association was found between CKD and DR or cataract in this group. This discrepancy may be explained by ethnic differences in genetic susceptibility, or the presence of other modifying risk factors such as BP, BMI or smoking that influence the manifestation of eye complications in CKD. Glaucoma showed a protective association with CKD in Malays, a finding that is not yet well understood. Studies suggest that lower intraocular pressure in CKD patients, due to fluid imbalances and other systemic factors, may play a role in glaucoma risk [27–29].

The strength of our study lies in the large, population-based study design, the availability of data on potential confounders, and the use of standardised protocols and assessments for eye diseases, which enhance the reliability of the findings. The limitations include the evaluation of CKD using a single measurement of serum creatinine and eGFR, which may have led to misclassification of CKD status. In addition, the number of incident events for individual eye diseases (*e.g.* glaucoma) was limited. Previous studies have reported a higher prevalence of vision-threatening retinal complications, such as DR, hypertensive retinopathy, retinal vein or artery occlusions, and glaucoma among patients on dialysis. However, we did not include this population. Future research should incorporate dialysis patients, given the substantial burden of eye diseases observed in this group [8,30,31].

## CONCLUSIONS

Our results suggest that CKD is associated with the incidence of VI and moderate/worse DR in Asian adults. Given the increasing prevalence of CKD worldwide, these findings highlight the importance of regular eye examinations for individuals with CKD to reduce the risk of VI and age-related eye diseases.

## Additional material


Online Supplementary Document
