# Supplementary material for: Impact of chronic kidney disease on the incidence of visual impairment and age-related eye diseases in a multi-ethnic Asian population
Source: J Glob Health. 2025 Nov 28;15:04316. doi: 10.7189/jogh.15.04316 (PMC12659799; doi:10.7189/jogh.15.04316)
Supplement: Online Supplementary Document [file jogh-15-04316-s001.pdf]

Supplement to: Liem Y, Vemula V, Lim CC, Chong CCY, Choo JCJ, Cheng CY, Sabanayagam C. Impact of chronic kidney disease on the incidence of visual impairment and age-related eye diseases in a multi-ethnic Asian population. J Glob Health. 2025;15:04316.

SUPPLEMENTARY MATERIALS

Table S1. STROBE Statement—Checklist of items that should be included in reports of cohort studies

	Item No	Recommendation	Included
**Title and abstract**	1	(*a*) Indicate the study's design with a commonly used term in the title or the abstract	Yes
		(*b*) Provide in the abstract an informative and balanced summary of what was done and what was found	Yes
**Introduction**			
Background/rationale	2	Explain the scientific background and rationale for the investigation being reported	Yes
Objectives	3	State specific objectives, including any prespecified hypotheses	Yes
**Methods**			
Study design	4	Present key elements of study design early in the paper	Yes
Setting	5	Describe the setting, locations, and relevant dates, including periods of recruitment, exposure, follow-up, and data collection	Yes
Participants	6	(*a*) Give the eligibility criteria, and the sources and methods of selection of participants. Describe methods of follow-up	Yes
		(*b*) For matched studies, give matching criteria and number of exposed and unexposed	N/A
Variables	7	Clearly define all outcomes, exposures, predictors, potential confounders, and effect modifiers. Give diagnostic criteria, if applicable	Yes
Data sources/ measurement	8*	For each variable of interest, give sources of data and details of methods of assessment (measurement). Describe comparability of assessment methods if there is more than one group	Yes
Bias	9	Describe any efforts to address potential sources of bias	Yes
Study size	10	Explain how the study size was arrived at	Yes

Quantitative variables	11	Explain how quantitative variables were handled in the analyses. If applicable, describe which groupings were chosen and why	Yes
Statistical methods	12	(*a*) Describe all statistical methods, including those used to control for confounding	Yes
		(*b*) Describe any methods used to examine subgroups and interactions	Yes (eGFR and ethnicity sub analyses)
		(*c*) Explain how missing data were addressed	Yes
		(*d*) If applicable, explain how loss to follow-up was addressed	N/A
		(*e*) Describe any sensitivity analyses	N/A
**Results**			
Participants	13*	(a) Report numbers of individuals at each stage of study—eg numbers potentially eligible, examined for eligibility, confirmed eligible, included in the study, completing follow-up, and analysed	Yes
		(b) Give reasons for non-participation at each stage	N/A
		(c) Consider use of a flow diagram	Yes (Flow diagram in the main text)
Descriptive data	14*	(a) Give characteristics of study participants (eg demographic, clinical, social) and information on exposures and potential confounders	Yes

		(b) Indicate number of participants with missing data for each variable of interest	Yes (Flow diagram in the main text)
		(c) Summarise follow-up time (eg, average and total amount)	Yes
Outcome data	15*	Report numbers of outcome events or summary measures over time	Yes
Main results	16	(*a*) Give unadjusted estimates and, if applicable, confounder-adjusted estimates and their precision (eg, 95% confidence interval). Make clear which confounders were adjusted for and why they were included	Yes
		(*b*) Report category boundaries when continuous variables were categorized	Yes
		(*c*) If relevant, consider translating estimates of relative risk into absolute risk for a meaningful time period	N/A
Other analyses	17	Report other analyses done—eg analyses of subgroups and interactions, and sensitivity analyses	Yes (eGFR and ethnicity subgroup analyses)
**Discussion**			
Key results	18	Summarise key results with reference to study objectives	Yes
Limitations	19	Discuss limitations of the study, taking into account sources of potential bias or imprecision. Discuss both direction and magnitude of any potential bias	Yes

Interpretation	20	Give a cautious overall interpretation of results considering objectives, limitations, multiplicity of analyses, results from similar studies, and other relevant evidence	Yes
Generalisability	21	Discuss the generalisability (external validity) of the study results	Yes
**Other information**			
Funding	22	Give the source of funding and the role of the funders for the present study and, if applicable, for the original study on which the present article is based	Yes

Table S2. Definitions and assessment criteria for eye diseases.

Eye disease	Definition	Criteria / Grading System
VI	Best-corrected visual acuity (BCVA) worse than 20/40 in the better eye	Severity categories:<br>Normal vision: BCVA ≥20/40 (logMAR ≤0.30)<br>Low vision: BCVA <20/40 but >20/200 (logMAR >0.30 to <1.00)<br>Blindness: BCVA ≤20/200 (logMAR ≥1.00
Any retinopathy	Characteristic lesions on retinal photographs	-
DR	Retinopathy present in participants with diabetes	Early Treatment Diabetic Retinopathy Study severity levels: <20 (no DR), 20 (minimal), 35 (mild), 43–47 (moderate), 53 (severe), 61–90 (proliferative)
AMD	Presence of early or late AMD	Wisconsin Age-Related Maculopathy Grading System on fundus photographs
Glaucoma	Both glaucomatous visual field loss and optic disc changes in ≥1 eye	Visual field loss: glaucoma hemifield test “outside normal limits” and cluster of three contiguous points at 5% level on pattern deviation plot
Cataract	Presence of lens opacity or prior cataract surgery	Lens Opacities Classification System III (LOCS III): nuclear, cortical, or posterior subcapsular or a history of cataract surgery.
URE	Improvement in visual acuity with refraction	≥0.2 logMAR (≥2 lines) improvement in best-corrected vs. presenting visual acuity in better eye

Abbreviations: VI, vision impairment; DR, diabetic retinopathy; AMD, age-related macular; degeneration; URE, under-corrected refractive error

Table S3. Incidence of eye diseases stratified by baseline CKD severity.

Ocular Conditions	CKD stage G1-G2	CKD stage G3a	CKD stage G3b-G5	p-value	p-trend
Any VI	142 (3.3)	43 (13.5)	21 (16.3)	<0.001	<0.001
Any eye disease	1466 (45.0)	56 (63.6)	24 (72.7)	<0.001	<0.001
Any retinopathy	276 (5.2)	21 (6.4)	10 (8.3)	0.2	0.09
DR	157 (14.4)	17 (14.5)	5 (8.9)	0.5	0.36
Moderate/worse DR	82 (6.3)	15 (10.5)	3 (4.9)	0.2	0.46
Any AMD	294 (5.4)	27 (8.0)	11 (8.1)	0.06	0.02
Glaucoma	105 (1.8)	9 (2.3)	2 (1.3)	0.7	0.93
Cataract	1487 (40.8)	71 (64.0)	28 (68.3)	<0.001	<0.001
URE	321 (9.1)	28 (10.2)	17 (15.3)	0.07	0.03

Abbreviations: CKD, chronic kidney disease; VI, vision impairment; DR, diabetic retinopathy; AMD, age-related macular; degeneration; URE, under-corrected refractive error. Data presented as the number (%) and p-values were calculated using Pearson's Chi-squared Test and Fisher’s Exact Test for those with subgroup numbers < 5.

Any VI: 4732 overall, 4285 CKD stage G1-2, 318 CKD stage G3a, 129 CKD stage G3b-5.

Any ocular disease: 3379 overall, 3258 CKD stage G1-2, 88 CKD stage G3a, 33 CKD stage G3b-5.

Any retinopathy: 5753 overall, 5302 CKD stage G1-2, 330 CKD stage G3a, 121 CKD stage G3b-5.

DR: 1262 overall, 1089 CKD stage G1-2, 117 CKD stage G3a, 56 CKD stage G3b -5.

Moderate/ worse DR: 1507 overall, 1303 CKD stage G1-2, 143 CKD stage G3a, 61 CKD stage G3b-5.

Any age-related macular degeneration: 5893 overall, 5420 CKD stage G1-2, 337 CKD stage G3a, 136 CKD stage G3b-5.

Glaucoma: 6299 overall, 5764 CKD stage G1-2, 383 CKD stage G3a, 152 CKD stage G3b-5.

Cataract: 3799 overall, 3647 CKD stage G1-2, 111 CKD stage G3a, 41 CKD stage G3b-5.

Under-corrected refractive error: 3918 overall, 3533 CKD stage G1-2, 274 CKD stage G3a, 111 CKD stage G3b-5.

Table S4. Associations of baseline eGFR with incidence VI and major ocular diseases

Ocular Conditions	Age, sex adjusted OR (95% CI)	p-value	Multivariable OR (95% CI)*	p-value
Any VI	1.11 (1.06-1.15)	<0.001	1.06 (1.01-1.11)	0.01
Any eye disease	1.05 (1.03-1.08)	<0.001	0.99 (0.96-1.02)	0.5
Any retinopathy	0.98 (0.95-1.02)	0.4	0.97 (0.93-1.01)	0.2
**DR	0.95 (0.91-1.00)	0.07	0.97 (0.92-1.03)	0.3
**Moderate/ worse DR	1.00 (0.94-1.07)	0.9	1.04 (0.97-1.11)	0.3
Any AMD	0.98 (0.95-1.02)	0.3	0.96 (0.92-1.00)	0.05
Glaucoma	0.95 (0.89-1.01)	0.08	0.95 (0.89-1.02)	0.1
Cataract	1.07 (1.04-1.09)	<0.001	1.00 (0.98-1.03)	0.8
URE	1.01 (0.98-1.05)	0.4	0.99 (0.95-1.02)	0.4

Abbreviations: eGFR, estimated glomerular filtration rate; OR, odd ratio; CI, confidence interval; VI, vision impairment; DR, diabetic retinopathy. AMD: age-related macular degeneration; URE, under-corrected refractive error.

* Adjusted for age, gender, ethnicity, diabetes status, and hypertension.

** Adjusted for age, gender, ethnicity, diabetes duration, and HbA1c.

Odds ratio of eGFR is per 5ml/min/1.73 m^2 decrease.

Figure S1. Incidence of VI by CKD severity

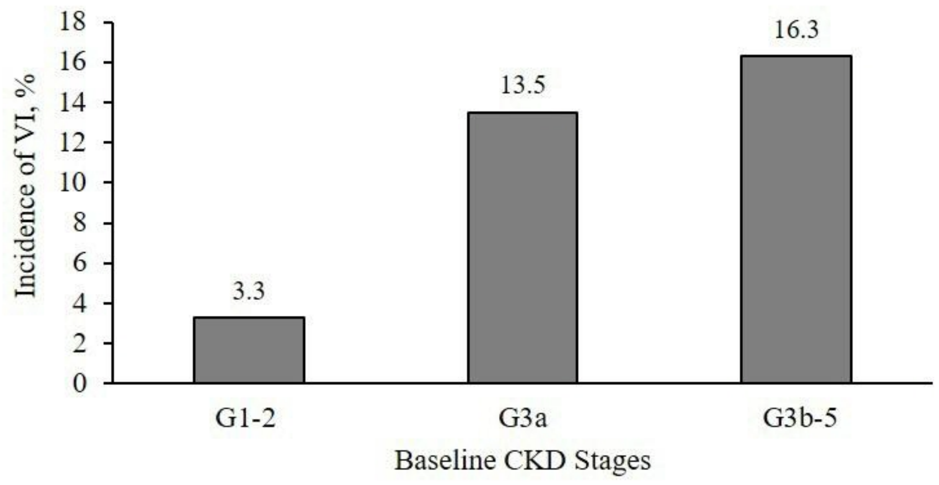

Abbreviations: VI, vision impairment.

P-value was calculated using Chi-squared Test. Overall, p-trend<0.001, signifying a significant association between CKD severity and the likelihood of VI.

Figure S2. Incidence of VI by baseline CKD status stratified by ethnicity

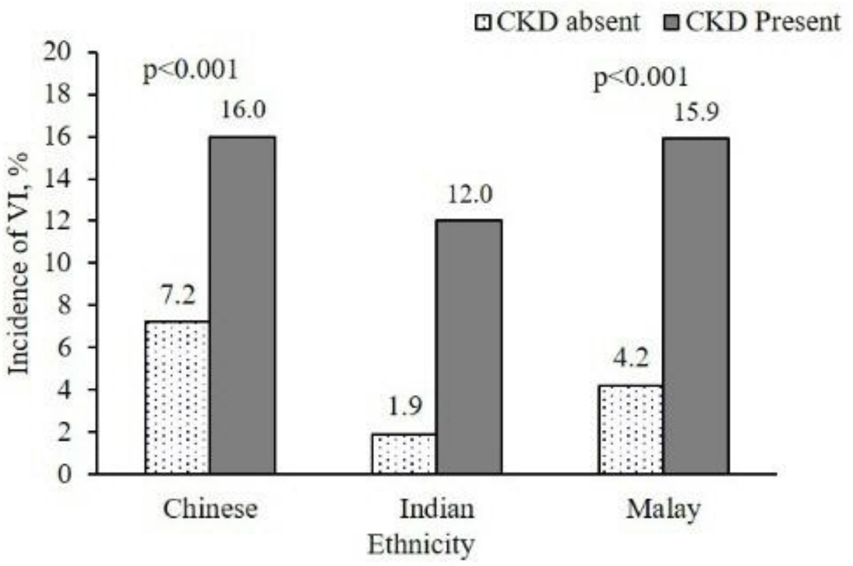

Abbreviations: CKD, chronic kidney disease; VI, vision impairment.

Data presented as the number (%) and p-values were calculated using Pearson's Chi-squared Test.
